# Interaction between Infection of Porphyromonas gingivalis, A Keystone Microbe of Oral Microbiome, and Serum Levels of Lutein/Zeaxanthin Is Associated with Risk for Age-related Macular Degeneration

**DOI:** 10.21203/rs.3.rs-6188207/v1

**Published:** 2025-05-06

**Authors:** Chung-Jung Chiu, Emily Chiu, Min-Lee Chang

**Affiliations:** The Forsyth Institute; The University of Vermont; Harvard Medical School

**Keywords:** microbiota, microbiome, periodontitis, Porphyromonas gingivalis, retina, age-related macular degeneration, epidemiology, risk factors, lutein, zeaxanthin, National Health and Nutrition Examination Survey (NHANES)

## Abstract

*Porphyromonas gingivalis (P. gingivalis)* functions as a catalyst bacterium in the development of periodontitis, and the serum antibody level against *P. gingivalis* is considered a surrogate marker for the activity level of periodontopathic microbiome. The chronic systemic inflammation induced by *P. gingivalis* elevates the risk of various systemic and neurodegenerative disorders, including atherosclerosis, diabetes, and Alzheimer’s disease. Although the connection between human microbiome and age-related macular degeneration (AMD) remains relatively unexplored, it is noteworthy that AMD shares risk factors and etiological mechanisms with diseases related to *P. gingivalis*. To investigate the potential association between periodontopathic microbiome and AMD occurrence, we conducted a candidate microbe approach case-control study. Our hypothesis was tested by examining the correlation between serum *P. gingivalis* immunoglobulin G (IgG) levels and AMD. Comparing the lowest IgG category (≤ 57 enzyme-linked immunosorbent assay units (EU)) with higher categories revealed escalating risks: the second higher category (58–65 EU) conferred almost a 30% increased risk (odds ratio (OR) = 1.28, 95% confidence interval (CI): 1.17 to 1.4), the third higher category (66–119 EU) conferred nearly a 60% increase (OR = 1.58, 95% CI: 1.46 to 1.72), and the highest category (> 119 EU) conveyed over a two-fold risk (OR = 2.04, 95% CI: 1.62 to 2.58) of early AMD. Aligning with the notion that the microbiome composition is significantly shaped by the host’s diet, our analysis indicates that sustaining elevated serum levels of lutein/zeaxanthin (≥ 0.35 μmol/L or ≥ 20 μg/dL) might potentially mitigate the *P. gingivalis*-related AMD risk by as much as 35% (P for interaction < 0.0001). Although the precise mechanism requires additional exploration, these findings suggest a connection between nutrition and oral microbiome, emphasizing their collective role in maintaining eye health.

**SIGNIFICANCE STATEMENT:**

While our oral microbiome may impact eye health, nutritional factors could play a modulatory role in mitigating the associated risk.

## INTRODUCTION

Mucosal surfaces, including the oral mucosa, harbor a dynamic and intricate microbial community known as the “microbiome,” holding significant implications for human health and disease ^1^. Periodontal disease, a prevalent disease in human population ^2,3^, is significantly attributed to *Porphyromonas gingivalis (P. gingivalis)* ^4,5^, a gram-negative anaerobe primarily residing in the oral cavity. Colonizing the subgingiva, *P. gingivalis* contributes to the formation of a destructive biofilm (dental plaque) within a multispecies microbe community, leading to alveolar bone loss ^6^. Despite its low abundance, *P. gingivalis* acts as a catalyst in periodontitis, reshaping the composition of the oral commensal microbiome into a dysbiotic state, accelerating microbiome-mediated bone-destructive periodontitis ^7^. Moreover, the chronic trickling of this bacterium into the systemic bloodstream triggers a systemic inflammatory response, elevating levels of various inflammatory mediators ^8^. This *P. gingivalis*-induced systemic inflammation is linked to increased risks of systemic diseases such as atherosclerosis, rheumatoid arthritis, metabolic disorders ^4,9–12^, and neurodegenerative diseases, including cognition impairment and Alzheimer’s disease ^13,14^. This microbe serves as a vivid example of how the microbiome can impact diverse aspects of human health and disease in locations distant from its original habitat in the body. Significantly, our previous studies indicate that specific patterns of oral microbiome are strongly associated with human health and diseases, with *P. gingivalis* playing a pivotal role in patterns affecting retinal eye health ^15,16^. Age-related macular degeneration (AMD), a neurodegenerative disease of the retina causing blindness in individuals aged 65 + ^17^, shares risk factors and etiological mechanisms with *P. gingivalis*-related diseases ^18^. Hence, there is a hypothesis that periodontopathic microbiome is linked to the occurrence of AMD. To explore this, a “candidate microbe approach, association study” was conducted, correlating serum *P. gingivalis* immunoglobulin G (IgG) with AMD in a matched case-control study using data from the Third National Health and Nutrition Examination Survey (NHANES-III), a representative sample of the US population. Additionally, investigations were carried out to determine if modifiable risk factors for AMD could influence the *P. gingivalis*-related risk.

## MATERIALS and METHODS

### Study cohort

The Third National Health and Nutrition Examination Survey (NHANES-III) was performed between 1988 and 1994 by the National Center for Health Statistics. It is a cross-sectional nationwide health survey of 33994 non-institutionalized US residents aged 2 months and older using a stratified multistage probability sampling design to sample a representative cohort of the US general population.

### Case and control definitions

During the second phase of NHANES-III enrollment (1991–1994), 9371 persons had serum analysis for immunoglobulin levels of *P. gingivalis* ^19^, with 2925 persons ≥ 55 years of age. Of these, 1933 persons had gradable bilateral fundus photography at the time of the complete examination. We excluded persons with history of diabetes, heart attack, stroke, cancer, and missing covariate information. Races other than non-Hispanic white, non-Hispanic black, Mexican-American and participants on immunomodulatory medications or corticosteroids were also excluded from our study. Among non-smokers, other tobacco product users such as chewing tobacco, cigar, and pipe and cotinine level > 15 ng/ml were also excluded. Among the remaining eligible 1070 persons, 174 persons were identified as early AMD cases and 12 persons as late AMD cases. Early AMD was defined as the presence of either soft drusen (≥ 63 μm, equivalent to Grade 3 drusen in the Wisconsin Age-related Maculopathy Grading System) ^20^ or any drusen type with areas of depigmentation or hypopigmentation of the retinal pigment epithelium (RPE) without any visibility of choroidal vessels or with increased retinal pigment in the macular area. Late AMD was defined as the presence of signs of exudative macular degeneration or geographic atrophy (sharply delineated roughly round or oval area of apparent absence of the RPE in which choroidal vessels are more visible than in surrounding areas). The intergrader and intragrader Kappa scores ranged from 0.62 to 0.83 for the NHANES-III AMD grading, indicating a good reliability ^21^. Among the remaining 884 non-AMD persons, we selected a series of control subjects by a random selection of one-by-one frequency matching in age, sex, and race such that the overall characteristics distributions of the controls resembled the overall characteristics distributions of the cases.

### Serum P. gingivalis immunoglobulin G

Serum *P. gingivalis* IgG indicates systemic response to this periodontal disease-causing pathogenic bacterium. The antibody measurement in the NHANES-III data set was reported in enzyme-linked immunosorbent assay (ELISA) units (EU) of IgG. The detailed measurement methods are previously described elsewhere (National Center for Health Statistics NHANES III Data Documentation. http://www.cdc.gov/nchs/data/nhanes/nhanes3/depp.pdf). To examine for possible dose-response relationships of *P. gingivalis* IgG and AMD risk, we retained the same categorization ranges of *P. gingivalis* IgG from previous report from the Atherosclerosis Risk in Communities Study (ARIC) ^22^, which had similar demographics to the NHANES-III subjects ^14^. The report showed a significant (*P* < 0.0001) relationship between periodontitis severity and *P. gingivalis* IgG with a mean *P. gingivalis* IgG for healthy individuals of 53.8 EU, mild periodontitis 60.9 EU, moderate periodontitis 69.4 EU and severe periodontitis 168.4 EU. The midpoint between each of these *P. gingivalis* IgG means was used to create cut-off points for the four *P. gingivalis* IgG groups: ≤57 EU (referent), 58–65 EU, 66–119 EU and > 119 EU (highest).

### Statistical methods

The following were considered as covariates in our analyses: age, sex, race, education level, smoking status, body mass index (BMI, computed from weight and height; Kg/m^2^), drinking alcohol (at least 12 drinks in the past 12 months), serum levels of C reactive protein (CRP), vitamin C, vitamin E, and lutein/zeaxanthin, and two clinical periodontal measurements (mean number of tooth sites that bled on probing [mBOP] and mean clinical attachment loss [mCAL]). Descriptive statistics for these covariates between cases and controls were calculated. To determine significance of differences, analysis of variance (ANOVA) for comparison of means of continuous variables and chi-square tests for categorical variables were used. We also examined the correlations between serum *P. gingivalis* IgG and these covariates using Spearman correlation coefficients, Mann-Whitney tests, or Kruskal-Wallis tests, as appropriate.

To evaluate the association between *P. gingivalis* IgG and AMD risk, logistic regression models were fitted by controlling for selected covariates. All analyses were performed using SAS^®^ SURVEYLOGISTIC procedure (version 9.3; SAS Institute Inc, Cary, NC), which takes into account of the complex sampling design used in NHANES-III and yields unbiased standard error (SEM) and confidence interval (CI) estimates. Odds ratios (ORs) were calculated by dividing the odds of AMD among persons in higher categories of serum levels of *P. gingivalis* IgG by the odds among persons in the lowest category of *P. gingivalis* IgG. We used *P* < 0.05 to denote statistical significance and all tests were two-sided.

This study involved only the secondary data analysis of existing US national databases that are publicly available and have been de-identified. This research qualified for exemption of institutional review board human subjects approval under 45 CFR 46.101(b) (4) as specified by the Federal Regulations for Protection of Human Research Subjects. Thus, this is an exempt study and there was no need for institutional review board approval from our institutions. This human observational study report was prepared to conform to the STROBE guidelines.

## RESULTS

Since our controls were matched with cases in age, sex, and race, it is not surprising that the distributions of these three covariates were not significantly different between cases and controls (Table 1). Probably due to this matching strategy, the distributions for the other covariates were not significantly different, either. However, serum *P. gingivalis* IgG categorical distributions showed significantly different (*P*<0.0001) between cases and controls, and cases tended to be in the higher IgG categorical levels than controls, and the vice versa.

In our bivariate analysis, age (*P*=0.007) and serum vitamin C level (*P*=0.004) were inversely correlated with serum *P. gingivalis* IgG level while serum vitamin E level (*P*=0.004) was positively correlated (Table 2). Male sex (*P*=0.01), non-Hispanic black (*P*<0.0001), lower levels of education (*P*=0.01), and former smokers (*P*=0.001) tended to have higher levels of serum *P. gingivalis* IgG. However, BMI, alcoholic intake, and serum levels of lutein/zeaxanthin and CRP were not significantly correlated with serum *P. gingivalis* IgG level.

Next, in the logistic analysis evaluating our primary interest of the association between serum *P. gingivalis* IgG level and risk for AMD, we used a hierarchical strategy in our model construction to examine the confounding effects from the covariates (Table 3). Starting from an age-adjusted model (Model 1), we stepwise included the other covariates; Model 2 additionally adjusted for demographic covariates, including sex, race, BMI and education; Model 3 additionally adjusted for habitual exposures, including smoking history and alcohol intake; Model 4 additionally adjusted for serum levels of nutrient covariates, including vitamin C, vitamin E, lutein/zeaxanthin, and CRP, and Model 5 additionally adjusted for two clinical periodontal measurements (mBOP and mCAL). As shown in Table 3, the OR and 95% CI for each serum *P. gingivalis* IgG categorical level in every higher hierarchical models were similar with the age-adjusted OR and 95% CI, which showed a significant trend (*P*=0.036) of increased risk by increasing serum *P. gingivalis* IgG level. Overall, compared with the lowest IgG category, the second higher category conferred a 20% increased risk for early AMD, the third higher category conferred a 40%–60%, and the highest (fourth) category conferred an over two-fold of risk. Because including more covariates in the models decreases the statistical power, the trend tests became less significant in higher hierarchical models, however, they were all within marginal significance (*P*<0.1). Similar results were noted when including the 12 late AMD cases (see Case and control definitions) in the analysis.

**Figure 1:**
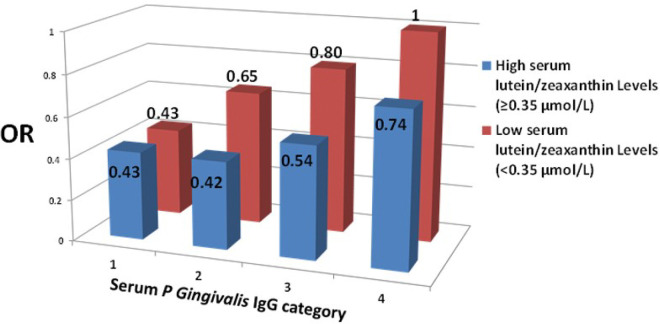
The *Porphyromonas gingivalis*-related AMD risk significantly (*P* for interaction<0.0001) varies by serum levels of lutein/zeaxanthin (≥0.35 μmol/L vs. <0.35 μmol/L).The four *P. gingivalis* IgG groups are ≤57 EU (category 1), 58–65 EU (category 2), 66–119 EU (category 3) and >119 EU (category 4). The ORs (95% CIs) for the four *P. gingivalis* IgG groups (from high to low) in the low serum lutein/zeaxanthin category are 1 (referent), 0.80 (0.45 to 1.42), 0.65 (0.40 to 1.07), and 0.43 (0.28 to 0.66), and they are 0.74 (0.48 to 1.15), 0.54 (0.37 to 0.80), 0.42 (0.28 to 0.63), and 0.43 (0.28 to 0.65) in the high serum lutein/zeaxanthin category, respectively. Abbreviation: OR, odds ratio; CI, confidence interval; IgG, immunoglobulin G*; EU,* enzyme-linked immunosorbent assay *unit*.

We further tried to evaluate if the effect of serum *P. gingivalis* IgG level on AMD risk varies by the status of modifiable risk factors for AMD, including smoking status (ever smokers vs. non-smokers), BMI (≥25 vs. <25 or ≥28 vs. <28 or ≥30 vs. <30 Kg/m^2^), and serum levels (higher vs. lower than the median) of vitamin C (median=39 mmol/L), vitamin E (median=23.5 μmol/L), and lutein/zeaxanthin (mediam=0.35 μmol/L). The results indicated that the *P. gingivalis*-related AMD risk significantly (*P* for interaction<0.0001) varies by serum levels of lutein/zeaxanthin (≥0.35 μmol/L vs. <0.35 μmol/L). Compared subjects in the low serum level of lutein/zeaxanthin with those in the high serum level, there is an up to 35.4% (=(0.65–0.42)/0.65) higher risk of AMD for serum *P. gingivalis* IgG category 2 (58–65 EU), 32.5% (=(0.80–0.54)/0.80) for category 3, and 26% (=(1–0.74)/1) for category 4 (Figure 1). In other words, higher serum lutein/zeaxanthin levels were protective against *P. gingivalis*-related AMD risk.

## DISCUSSION

Traditionally, microbiology in the context of human health primarily concentrated on local effects. However, our study has revealed a notable shift in perspective, demonstrating a positive association between the serum signature of *P. gingivalis* and the risk of AMD. This contributes to the growing body of evidence suggesting that the microbiome within the human body can exert influences on distant tissues and organs. Additionally, aligning with the notion that the microbiome composition is significantly shaped by host’s diet, our findings indicate that elevated serum levels of lutein/zeaxanthin offer a protective effect against the *P. gingivalis*-related AMD risk.

To date, only few studies have been published that explores the correlation between periodontitis and AMD ^23–25^. Although pooled analysis suggested that periodontitis patients may have a higher risk of AMD ^23,24^, bias assessment and power analysis indicated that the association remains debatable ^25^.

As part of the cross-sectional Finnish national population-based Health 2000 Survey ^26^, 1751 individuals aged 30 years or older were included in the study, consisting of 54 individuals with degenerative fundus changes (AMD group) and 1,697 individuals free of AMD (non-AMD group). In their univariate analysis comparing the AMD group with the non-AMD group, Karesvuo et al. identified a significant difference in the proportion of individuals with alveolar bone loss among males and a significant difference in the number of teeth among females. However, likely due to insufficient case numbers and control selection, no significant difference was found between the AMD group and the non-AMD group in terms of the proportion of carriage of salivary periodontopathic bacteria, including *P. gingivalis*. Following multivariate adjustment for various factors such as age, diabetic status, systolic blood pressure, education, smoking, and the carriage of salivary bacteria, only alveolar bone loss remained significantly associated with the risk of AMD among males.

While previous research has proposed infection as a potential risk factor for AMD ^27^, and *P. gingivalis* has been linked to various human neurodegenerative disorders ^13,14^, our study is the first to establish a significant relationship between *P. gingivalis* and AMD. Notably, *P. gingivalis* is not limited to the oral cavity, as it also inhabits other sites within the human body. The ubiquitously expressed transglutaminase 2 (TG2) plays a crucial role in *P. gingivalis* adherence to host cells ^6^, with periodontitis being its sole known clinical manifestation in situ. Furthermore, *P. gingivalis* acts as a catalyst in periodontopathic microbiome, and the serum *P. gingivalis* IgG level has been demonstrated to closely correlate with the severity of periodontitis ^7,22^. As a result, the serum *P. gingivalis* IgG level can function as a surrogate marker for the activity level of periodontopathic microbiome in the oral cavity ^4^.

In contrast to most pathogenic bacteria that typically induce severe inflammation and outcompete native bacteria, *P. gingivalis* establishes colonization at low levels and functions as a “catalyst” to foster a pathogenic microbiome (pathobionts). Studies in a murine periodontal model have demonstrated that even at low numbers, the introduction of *P. gingivalis* into the oral microbiome community significantly accelerates pathological alveolar bone loss ^28^. However, since *P. gingivalis* alone fails to induce periodontitis, the hypothesis arises that *P. gingivalis* exerts its bone-destructive role in collaboration with other dysbiotic bacteria. Mechanistic investigations indicate that *P. gingivalis* colonization in the oral cavity disrupts the host immune system and induces changes in the quantity and composition of the oral commensal microbiome. This occurs through the secretion of gingipain, a complement component 5 (C5) convertase-like enzyme. Gingipain generates elevated levels of locally active C5a, leading to C5aR activation, triggering inflammation while simultaneously inhibiting the killing capacity of leukocytes and suppressing the expression of chemokines. Studies further highlight the significance of the complement pathway in *P. gingivalis*-related pathogenesis, proposing the targeting of C3 as a potential treatment strategy for periodontitis ^29^. Interestingly, it is well-documented that the activation of C3 and the generation of excessive quantities of C5a and C5b-C9 play a significant role in the pathogenesis of AMD ^30^. While it has been established that the inflammatory and immune response triggered by *P. gingivalis* has both local and systemic effects ^4,9–12^, the impact of gingipain, C5 activation, and the dysbiotic microbiome induced by *P. gingivalis* on the retina is yet to be determined.

If an established etiological relationship between *P. gingivalis* and AMD is confirmed, AMD could join the ranks of diseases—such as obesity, metabolic disorders, and inflammatory bowel diseases—that have been shown to be transmittable through the transfer of dysbiotic microbiome ^31^. In such a scenario, the management of *P. gingivalis*-related AMD risk could involve the elimination of *P. gingivalis* from the oral cavity. However, it is worth noting that the composition of the microbiome is highly susceptible to changes influenced by the host microenvironment and diet ^32^. Our analysis (Figure 1) also suggests that maintaining a higher serum level of lutein/zeaxanthin (≥0.35 μmol/L or ≥20 μg/dL), possibly through a healthy diet or the use of the Age-Related Eye Disease Study 2 (AREDS2) supplement ^33–36^, could help modulate the *P. gingivalis*-related AMD risk. Although studies have demonstrated that lower serum levels of various carotenoids, including zeaxanthin, increase the risk of periodontitis ^37^, and supplemental lutein/zeaxanthin has been shown to be protective against AMD ^36^, it remains to be determined if lutein/zeaxanthin has a direct impact on the *P. gingivalis*-driven microbiome.

This study boasts several strengths, including its design as a matched case-control study within a representative cohort of the US population. The standardized collection of risk factor information and the use of photographic grading for maculopathy are additional strengths, aiming to minimize the impact of confounding factors and misclassifications. However, it is important to acknowledge certain limitations. The restricted number of AMD cases in our study resulted in insufficient sample sizes for certain analyses. For instance, in our interaction analyses, we only had a sufficient sample size to assess the relationship with serum levels of lutein/zeaxanthin. The cross-sectional nature of the study also poses a limitation in terms of defining temporality. Nevertheless, it’s worth noting that serum *P. gingivalis* IgG is considered to reflect chronic, intermittent exposure ^14^, and the average age of onset for periodontitis is notably younger than that for AMD ^2^. Additionally, serum levels of lutein/zeaxanthin are considered to reflect the long-term intake of these nutrients ^36^.

In conclusion, our study has unveiled a novel association between exposure to *P. gingivalis*, serum lutein/zeaxanthin levels, and the risk for AMD. Although the intricate mechanisms underlying this relationship require further investigation, our findings have the potential to significantly influence therapeutic and preventive strategies for AMD. This is particularly noteworthy given the high prevalence of *P. gingivalis* in the human population.

## Figures and Tables

**Table 1.** Comparisons in characteristics distributions between cases and frequency-matched controls in age, sex, and race.

Characteristics	Controls^a^	Cases^a^	*P* value^b^
	N=174	N=174	
**Age yrs (Mean (SEM))**	68.1 (0.6)	69.3 (0.2)	0.30
**Male (N (%))**	67 (46.1)	67 (42.5)	0.09
**Race (N (%))**			
*Non-Hispanic white*	108 (92.1)	108 (90.6)	0.17
*Non-Hispanic black*	27 (5.5)	27 (6.4)	
*Mexican-American*	39 (2.5)	39 (3.0)	
**BMI Kg/m^2^ (Mean (SEM))**	26.7 (0.1)	27.8 (0.3)	0.22
**Education (N (%))**			
*<12 yrs*	90 (29.9)	90 (32.9)	0.32
*12 yrs*	48 (37.1)	44 (32.5)	
*>12 yrs*	36 (32.9)	40 (34.5)	
**Smoking history (N (%))**			
*Non-smokers*	95 (52.1)	95 (51.2)	0.96
*Former smokers*	50 (38.7)	57 (39.4)	
*Active smokers*	29 (9.2)	22 (9.5)	
**At least 12 drinks in the past 12 months**			
*No*	119 (61.4)	120 (60.5)	0.58
*Yes*	55 (38.6)	54 (39.5)	
**Serum CRP level mg/dL (Mean (SEM))**	0.4 (0.01)	0.5 (0.004)	0.45
**Serum vitamin C mmol/L (Mean (SEM))**	56.8 (0.5)	52.1 (0.8)	0.28
**Serum vitamin E μmol/L (Mean (SEM))**	31.9 (0.5)	33.9 (0.2)	0.28
**Serum lutein/ zeaxanthin μmol/L (Mean (SEM))**	0.5 (0.01)	0.4 (0.01)	0.19
***Porphyromonas gingivalis* IgG EU**			
*≤57 EU (N (%))*	10 (8.0)	12 (5.8)	<0.0001
*58–65 EU (N (%))*	34 (21.7)	25 (18.4)	
*66–119 EU (N (%))*	95 (58.0)	95 (57.5)	
*>119 EU (N (%))*	35 (12.3)	42 (18.2)	

a For categorical variables, sample sizes (N) are raw numbers while the percentages (%) are weighted for the sampling design used in the Third National Health and Nutrition Examination Survey (NHANES-NI).

b Analysis of variance (*ANOVA*) was used for statistical tests of significance for continuous variables while Wald chi-squared test was used for all other categorical measures.

Abbreviation: BMI, body mass index; CRP, C reactive protein; IgG, immunoglobulin G; *EU*, enzyme-linked immunosorbent assay *unit*; SEM, standard error.

**Table 2.** Bivariate associations between serum *Porphyromonas gingivalis* immunoglobulin G concentrations and covariates.

Continuous covariates	N	Spearman correlation coefficient with *P. gingivalis* IgG (EU)	*P* value
**Age yrs**	348	−0.145	0.007
**BMI Kg/m^2^**	348	0.068	0.21
**Serum CRP level mg/dL**	348	0.081	0.13
**Serum vitamin C mmol/L**	348	−0.153	0.004
**Serum vitamin E μmol/L**	348	0.154	0.004
**Serum lutein/zeaxanthin μmol/L**	348	0.006	0.91
Categorical covariates	N	*P. gingivalis* IgG (EU), median [IQR]	*P* value^a^
**Sex**			
**Male**	134	80 [66–107]	0.01
**Female**	214	70 [64–95]	
**Race**			
***Non-Hispanic white***	216	73 [64–93]	<0.0001
***Non-Hispanic black***	54	102 [76–219]	
***Mexican-American***	78	100 [78–174]	
**Education**			
***<12 yrs***	180	76 [65–114]	0.01
***12 yrs***	92	74 [65–87]	
***>12 yrs***	76	72 [64–91]	
**Smoking history**			
***Non-smokers***	190	69 [64–96]	0.001
***Former smokers***	107	82 [68–109]	
***Active smokers***	51	70 [67–82]	
**At least 12 drinks in the past 12 months**			
***No***	239	74 [66–95]	0.51
***Yes***	109	73 [64–109]	
***Porphyromonas gingivalis* IgG category**			
***≤57 EU***	22	57 [56–57]	<0.0001
***58–65 EU***	59	62 [61–64]	
***66–119 EU***	190	77 [70–91]	
***>119 EU***	77	193 [151–449]	

a P-values were obtained by Mann-Whitney test for variables with 2 categories, and from Kruskal-Wallis test for variables with 3 or more categories.

Abbreviation: BMI, body mass index; CRP, C reactive protein; IgG, immunoglobulin G; *EU*, enzyme-linked immunosorbent assay *unit*; IQR, inter quartile range.

**Table 3.** Logistic analysis relating serum *Porphyromonas gingivalis* immunoglobulin G levels to risk for age-related macular degeneration.

Serum *P. gingivalis* IgG level (EU)	Adjusted OR (95% CI)^a^				
	Model 1	Model 2	Model 3	Model 4	Model 5
**≤57 (referent)**	1	1	1	1	1
**58–65**	1.19 (1.05 to 1.35)	1.23 (1.07 to 1.41)	1.24 (1.07 to 1.43)	1.27 (1.16 to 1.38)	1.28 (1.17 to 1.40)
**66–118**	1.44 (1.31 to 1.58)	1.48 (1.27 to 1.73)	1.51 (1.32 to 1.73)	1.55 (1.42 to 1.68)	1.58 (1.46 to 1.72)
**>119**	2.02 (1.66 to 2.46)	2.07 (1.67 to 2.55)	2.03 (1.52 to 2.72)	2.12 (1.68 to 2.66)	2.04 (1.62 to 2.58)
***P* for trend**	0.04	0.05	0.06	0.06	0.09

a Model 1: adjusted for age; Model 2: Model 1 additionally adjusted for sex, race, BMI and education; Model 3: Model 2 additionally adjusted for smoking history and alcohol intake; Model 4: Model 3 additionally adjusted for serum vitamin C, E, lutein/xeaxanthin, and CRP levels; Model 5: Model 4 additionally adjusted for two clinical periodontal measurements, mBOP and mCAL.

Abbreviation: OR, odds ratio; CI, confidence interval; CRP, C reactive protein; IgG, immunoglobulin G; *EU*, enzyme-linked immunosorbent assay *unit;* mBOP, mean number of tooth sites that bled on probing; mCAL, mean clinical attachment loss.

## Data Availability

The data used in this study is freely available for download by the public at: https://wwwn.cdc.gov/nchs/nhanes/nhanes3/default.aspx.
